# Effective protection of ZF2001 against the SARS-CoV-2 Delta variant in lethal K18-hACE2 mice

**DOI:** 10.1186/s12985-022-01818-x

**Published:** 2022-05-20

**Authors:** Lianlian Bian, Yu Bai, Fan Gao, Mingchen Liu, Qian He, Xing Wu, Qunying Mao, Miao Xu, Zhenglun Liang

**Affiliations:** Division of Hepatitis and Enterovirus Vaccines, NHC Key Laboratory of Research on Quality and Standardization of Biotech Products, and NMPA Key Laboratory for Quality Research and Evaluation of Biological Products, Institute of Biological Products, National Institutes for Food and Drug Control, Beijing, China

**Keywords:** ZF2001, Protection, SARS-CoV-2 Delta variant, K18-hACE2 mice

## Abstract

To investigate the protective efficacy and mechanism of ZF2001 (a protein subunit vaccine with conditional approval in China) to SARS-CoV-2 Delta variant-induced severe pneumonia, the lethal challenge model of K18-hACE2 transgenic mice was used in this study. An inactivated-virus vaccine at the research and development stage (abbreviated as RDINA) was compared to ZF2001. We found that ZF2001 and RDINA could provide the protective effect against Delta variant-induced severe cases, as measured by the improved survival rates, the reduced virus loads, the alleviated lung histopathology and the high neutralizing antibody geomean titers, compared to aluminum adjuvant group. To prevent and control Omicron or other variant epidemics, further improvements in vaccine design and compatibilities with the novel adjuvant are required to achieve better immunogenicity.

**Supplementary Information:**

The online version contains supplementary material available at 10.1186/s12985-022-01818-x.

## Introduction

During the past year, the Severe Acute Respiratory Syndrome Coronavirus-2 (SARS-CoV-2) has continued to mutate into several variants. Among them, the Delta variant (B.1.617.2), which showed stronger transmission and partly immune escape ability, was defined as a variant of concern (VOC) by the World Health Organization (WHO). Recently, several imported outbreaks caused by Delta variant in China indicated that this VOC is still a serious challenge to some countries or regions with the effective epidemic prevention and control [1]. The published data indicated that recombinant protein vaccines, which account for the largest proportion of COVID-19 vaccine candidates according to WHO statistics, still provide effective protection against the Delta variant in clinical trials [2, 3]. In China, among the recombinant protein vaccines developed by domestic companies, ZF2001 was the first one to start and complete phase III clinical trials, and has been administered for nearly 200 million doses. Reports showed that the overall VE of ZF2001 against Delta variant is 78% by the phase III clinical trials [4, 5].

Delta variant could present the higher pathogenicity, compared to other variants. The reason is able to mediate highly efficient syncytium formation, the immune evasion, the higher replication and spike-mediated entry than wild-type (WT) strain [6]. To investigate the protective efficacy and mechanism of ZF2001 to Delta variant-induced severe pneumonia, the lethal challenge model of K18-hACE2 transgenic mice (established animal model) [7], which can present severe pneumonia post challenge with the Delta variant, was used in this study. An inactivated-virus vaccine at the research and development stage (abbreviated as RDINA) was compared to ZF2001. This study provides the basis for the application of ZF2001 against SARS-CoV-2 variants as well as the research and development of the next-generation vaccines.

## Materials and methods

### Biosafety and ethics approval and consent to participate

All experiments with live SARS-CoV-2 were performed in a biosafety level 3 (ABSL3) facility at the Wuhan Institute of Biological Products Co. LTD. All research staff has been trained and qualified in experimental operations. All animal procedures were approved by the Ethics Committee on Laboratory Animals of the Wuhan Institute of Biological Products Co. LTD. The number of ethical permissions for this experiment was WIBP-AII442021005.

### Virus

SARS-CoV-2 wild-type (WT, GenBank: MN996528.1) and Delta variant (GenBank: OK091006.1) isolates were propagated in Vero cells in DMEM (Invitrogen, Waltham, MA, USA) containing 10% fetal bovine serum (FBS), 100 IU/mL penicillin, and 100 μg/mL streptomycin, and incubated at 37 °C as previously described [8]. The CCID_50_ of the WT was 10^7.04^/mL and the CCID_50_ of the Delta variant was 10^5.5^/mL.

### Animal study

**Fig. 1:**
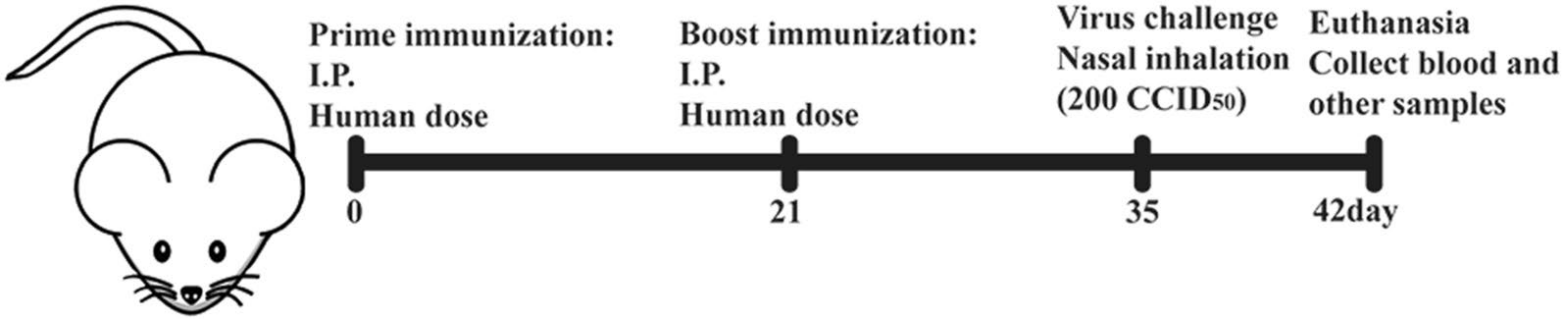
Procedure of the animal model study. I.P, intraperitoneal injection

Specific pathogen-free, 20 g K18-hACE2 transgenic mice (5 weeks old) were obtained from Beijing Vital River Laboratory Animal Technology Co., Ltd. (China). Thirty-six mice (18 females and 18 males) were acclimated for 2 days before the experiments to adapt to the environment and reduce stress. All mice were equally and randomly divided into six groups: aluminum adjuvant (Al) + WT (control group), RDINA + WT, ZF2001 + WT, Al + Delta, RDINA + Delta, and ZF2001 + Delta. Human doses of ZF2001 [containing 25 μg receptor binding domain (RBD)] and RDINA (containing 5 μg inactivate virus particles) were used to immunize mice. Aluminum hydroxide adjuvant (0.25 mg) was used as the negative control. The detailed experimental procedure is illustrated in Fig. 1. Mice in the six groups were weighed, and survival was monitored until the end of the experiment. Published research indicated that K18-hACE2 transgenic mice showed severe medical condition 7 days post-SARS-CoV-2 infection, and the severity degree on this day was representative [9]. Thus, mice were euthanized, and samples were collected 7 days post-SARS-CoV-2 challenge for the following experiments.

### Histopathological examination

The lungs were fixed in 4% paraformaldehyde, embedded in paraffin, sectioned, and stained using hematoxylin and eosin (H&E) for histopathological analysis. The results were confirmed by an experienced and qualified pathologist. Alveolar and tracheal structural damage, epithelial cell necrosis and shed, lung interstitial thickness, hyperemia, hemorrhage, and inflammatory cell infiltration were scored on a 0- to 4-point scale as follows: no pathological change = 0; pathological change in 0–25% of the field = 1; pathological change in 25–50% of the field = 2; pathological change in 50–75% of the field = 3; and pathological change in 75–100% of the field = 4 [10].

### Determination of SARS-CoV-2 gene copies by qRT-PCR

Total RNA was extracted from mouse lung tissues using the Nucleic Acid Isolation Kit (DAAN GENE, Guangzhou, China). The qRT-PCR was then performed using the Novel Coronavirus 2019-NCOV Nucleic Acid Detection Kit (DAAN GENE, Guangzhou, China).

### Determination of serum neutralizing antibody (Nab) titers

The levels of Nab titers against SARS-CoV-2 were measured using the WT and Delta variant, and the results were expressed as GMTs. Antibody titers were transformed into log_2_ values to calculate geometric means and 95% confidence intervals (CIs). The experiment was performed following a previously reported protocol [11].

### Statistical analyses

Statistical analyses were performed using GraphPad Prism 6.0 (https://www.graphpad.com). Statistical significance was evaluated for at least three replicates using the Tukey's multiple comparison test. Statistical analyses of the survival curves were performed using log-rank tests. Pearson’s correlation analysis was performed to calculate the relationship between Nab titers and survival rates.

## Result and discussion

**Fig. 2:**
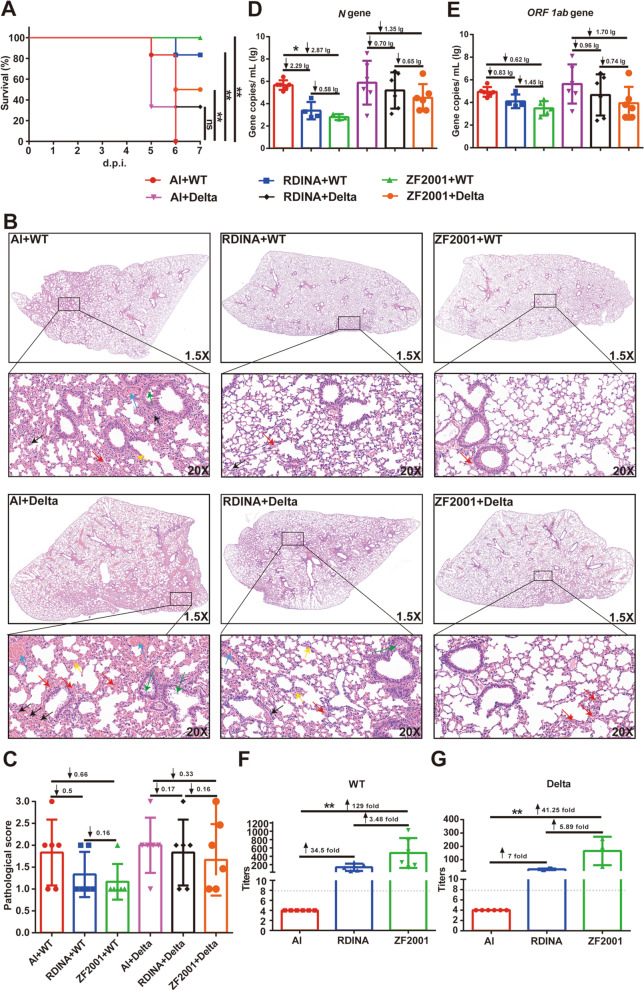
Survival curves, lung histopathology, virus gene copy numbers in lungs, and Nab titers of Al + WT, RDINA + WT, ZF2001 + WT, Al + Delta, RDINA + Delta, and ZF2001 + Delta groups. **a** Survival curves. **b** Lung histopathology (inflammatory cell infiltration indicated by black arrows, lung interstitial thickness indicated by yellow arrows, hyperemia indicated by blue arrows, hemorrhages indicated by red arrows, and epithelial cell necrosis and shed indicated by green arrows). **c** Histopathological score. **d, e** SARS-CoV-2N and *ORF 1ab* gene copy numbers. **f, g** Nab titers for WT and Delta variant. Antibody titers were transformed into log_2_ values to calculate geometric means and 95% confidence intervals. d.p.i, days post infection. ↓, decrease. ↑, increase. lg, log_10_. * = P < 0.05, ** = P < 0.01. ns, non-significance

In the Al immunized groups, all mice died 5 or 6 days after challenge with the WT or Delta variant of SARS-CoV-2, indicating that the challenge doses used in this experiment could cause high mortality in mice. For the ZF2001 immunized groups, the survival rates of mice were 100% and 50% post WT and Delta variant challenge, respectively, which were significantly higher than those of the Al-immunized groups (P < 0.01). For the RDINA immunized groups, mice survival rates were 83% and 33% post WT and Delta variant challenge, respectively (Fig. 2a). In addition, compared with the Al-immunized groups, less weight loss was observed in both the ZF2001- and RDINA-immunized groups from 4 days post-SARS-CoV-2 challenge (Additional file 1: Figure S1). The above results indicated that both ZF2001 and RDINA could provide the protection effect for the lethal mouse model caused by WT or Delta variant challenge, although survival rates against the Delta variant were lower than those against the WT for both vaccines.

Lung histopathological analysis showed severe alveolar and bronchus structural damage, lung interstitial thickness, epithelial cell necrosis and shedding, hyperemia, hemorrhages, and inflammatory cell infiltration in the Al-immunized groups after WT or Delta variant challenge. This indicated that both strains could cause severe interstitial pneumonia, lung consolidation, and injury in K18-hACE2 mice, which was similar to the pathological changes observed in humans infected with SARS-CoV-2 [12]. However, the severity of pneumonia, lung consolidation, and injury were alleviated in the ZF2001-and RDINA-immunized groups after the challenge with either strain (Fig. 2b, c). Compared to other existing models, the K18-hACE2 transgenic mouse model seems to be the most sensitive to SARS-CoV-2. This might be due to the K18 promoter exhibits the higher promoter activity or gene expression of ACE2 compared with other promoters [7]. As so, this mouse model may be used to better observe the protective effect of vaccines against the Delta variant and other VOC-induced severe pneumonia.

To determine viral loads in the lungs, quantitative real-time polymerase chain reaction (qRT-PCR) was used to detect SARS-CoV-2N and *ORF 1ab* gene copies. The results showed that *N* gene copies in the ZF2001- and RDINA-immunized groups decreased by 2.87 and 2.29 lg/mL compared to Al-immunized groups when challenged by the WT strain, and by 0.70 and 1.35 lg/mL when challenged by the Delta variant. In addition, *ORF 1ab* gene copies in the ZF2001- and RDINA-immunized groups were decreased by 0.83 and 0.62 lg/ mL compared to Al-immunized groups post infected with the WT strain, and by 0.96 and 1.70 lg/ mL when challenged by the Delta variant (Fig. 2d, e). The results suggested that ZF2001 and RDINA vaccines could reduce the replication of both strains in mouse lungs, while the reduction was lower for the Delta variant than for the WT.

David et al*.* found a remarkably strong non-linear relationship between the mean neutralization level and reported protection across the different vaccines in clinical trials [13]. Serum Nab titers were measured after the virus challenge. For the WT strain, Nab geomean titers (GMTs) of mice immunized with ZF2001 and RDINA were 369 and 114 (Fig. 2f), and survival rates were 100% and 83%, respectively. For the Delta variant, Nab GMTs of mice immunized with ZF2001 and RDINA were 133 and 28 (Fig. 2g), and survival rates were 50% and 33%, respectively. The correlation value (*r* = 0.8319) suggested that there might be a non-linear, positive relationship between Nab GMTs and the protective effects of vaccines in this study (Additional file 2: Figure S2). Above results indicated that Nab induced by ZF2001 and RDINA play the protective roles against the SARS-CoV-2 WT and Delta variant.

The RBD of WT SARS-CoV-2 was used as antigen in ZF2001 vaccine (Additional file 3). The published researches indicated that the RBD region has a potential ability that produces broad-spectrum Nabs against multiple SARS-CoV-2 variants [14–16]. For example, yeast-produced RBD-based recombinant protein vaccines could elicit broadly neutralizing antibodies and durable protective immunity against SARS-CoV-2, and variants B.1.1.7 and B.1.351 infection [14]. The published study found that glycan engineering of the SARS-CoV-2 RBD elicits cross-neutralizing antibodies for SARS-related viruses, also demonstrating that RBD has a potential ability to produces broad-spectrum vaccines against multiple SARS-related viruses [17]. The result of ZF2001 providing the neutralizing activity against Delta variant hinted that further optimization for adjuvants and selection of more suitable conserved sequences in RBD might be performed for this vaccine to produce the broad-spectrum Nab against variants.

The publish review indicated that subunit protein vaccines have good safety profiles and can be produced without specialized facilities like biosafety level 3 (BSL-3) workshop. And adjuvants can be added to the formulation to bolster immunogenicity. In contrast, inactivated vaccines also have good safety profiles, but it needed BSL-3 workshop facilities. Both of subunit and inactivated vaccines cannot adequately induce T cell immune response [11].

In summary, both SARS-CoV-2 strains (WT and Delta variant) caused severe interstitial pneumonia in K18-hACE2 mice. The protective effect of both vaccines on the lethal model suggested that ZF001 and RDINA could provide an effective protection on Delta variant-induced severe cases. To prevent and control Omicron or other variant epidemics, further improvements in vaccine design and compatibilities with the novel adjuvant are required to achieve better immunogenicity.

## Supplementary Information

**Additional file 1**. Mouse body weight changes in the Al + WT, RDINA + WT, ZF2001 + WT, Al + Delta, RDINA + Delta and ZF2001 + Delta groups.

**Additional file 2**. Correlation plot between Nab titers and survival rates.

**Additional file 3**. The information of ZF2001 and RDINA.

## Data Availability

All data generated or analyzed during this study are included in this published article.

## Abbreviations

SARS-CoV-2: Severe Acute Respiratory Syndrome Coronavirus-2
VOC: Variant of concern
WHO: World Health Organization
WT: Wild-type
Nab: Neutralizing antibody
Al: Aluminum adjuvant
qRT-PCR: Quantitative real-time polymerase chain reaction
GMTs: Nab geomean titers
RBD: Receptor binding domain
