# Supplementary figures and images for: Effective protection of ZF2001 against the SARS-CoV-2 Delta variant in lethal K18-hACE2 mice
Source: Virol J. 2022 May 20;19:86. doi: 10.1186/s12985-022-01818-x (PMC9122244; doi:10.1186/s12985-022-01818-x)

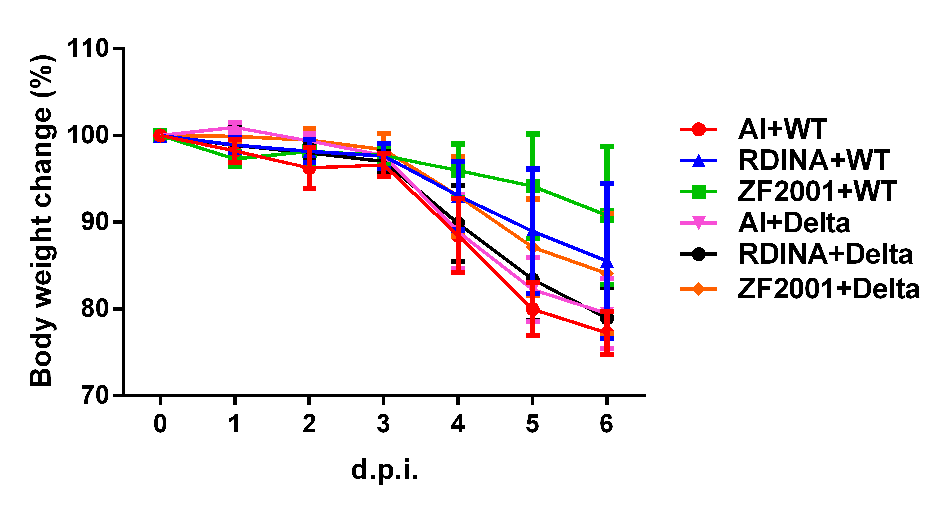

Supplement: Supplementary file 1 — Additional file 1. Mouse body weight changes in the Al + WT, RDINA + WT, ZF2001 + WT, Al + Delta, RDINA + Delta and ZF2001 + Delta groups. [file 12985_2022_1818_MOESM1_ESM.tif]

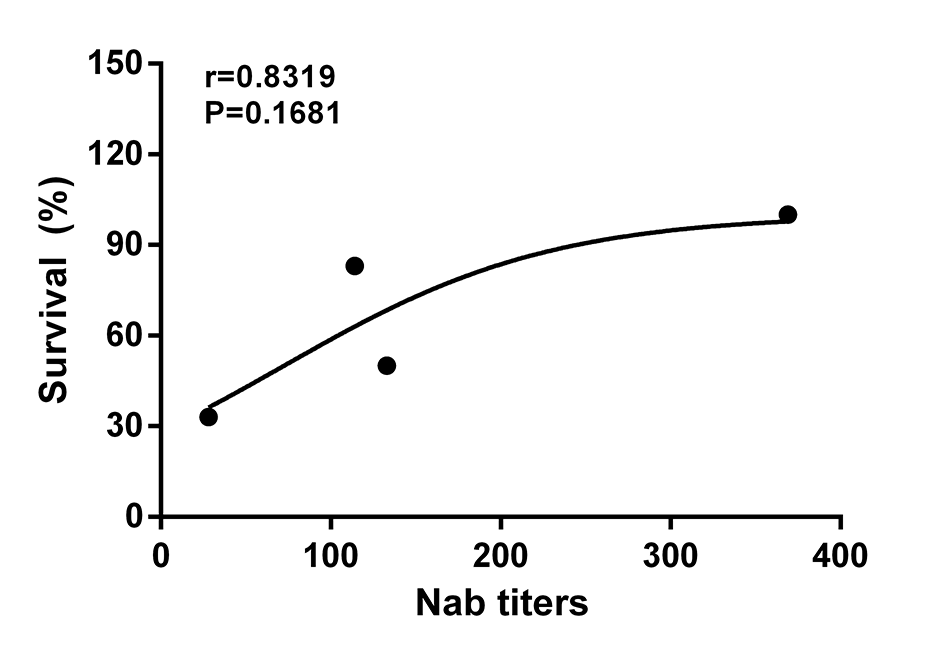

Supplement: Supplementary file 2 — Additional file 2. Correlation plot between Nab titers and survival rates. [file 12985_2022_1818_MOESM2_ESM.tif]
