# Supplementary material for: Effective protection of ZF2001 against the SARS-CoV-2 Delta variant in lethal K18-hACE2 mice
Source: Virol J. 2022 May 20;19:86. doi: 10.1186/s12985-022-01818-x (PMC9122244; doi:10.1186/s12985-022-01818-x)
Supplement: Supplementary file 3 — Additional file 3. The information of ZF2001 and RDINA. [file 12985_2022_1818_MOESM3_ESM.docx]

**Supplementary Information**

**The information of ZF2001 and RDINA**

ZF2001, produced by Anhui Zhifei Longcom Biologic Pharmacy Co., Ltd., is a kind of protein subunit vaccine with conditional approval in China. RBD of SARS-CoV-2 wide type strain, expressed by CHO cell lines, was used as antigen. And aluminum hydroxide was used as adjuvant for it. We can get more information in the reference “*A Universal Design of Betacoronavirus Vaccines against COVID-19, MERS, and SARS*” and the website (<http://www.zflongkema.com/product/ybcp/>).

RDINA was an inactivated-virus vaccine at the research and development stage. SARS-CoV-2 wide type strain was inactivated with β-propiolactone, followed by chromatography purification. Aluminum hydroxide was also used as adjuvant for it.

**Figure. S1.** Mouse body weight changes in the Al + WT, RDINA + WT, ZF2001 + WT, Al + Delta, RDINA + Delta and ZF2001 + Delta groups.

**Figure. S2.** Correlation plot between Nab titers and survival rates.
